# Development and validation of a machine learning model to predict time to renal replacement therapy in patients with chronic kidney disease

**DOI:** 10.1186/s12882-024-03527-9

**Published:** 2024-03-16

**Authors:** Jun Okita, Takeshi Nakata, Hiroki Uchida, Akiko Kudo, Akihiro Fukuda, Tamio Ueno, Masato Tanigawa, Noboru Sato, Hirotaka Shibata

**Affiliations:** 1 Department of Endocrinology, Metabolism, Rheumatology and Nephrology, Faculty of Medicine, Oita University, 8795593 1-1 idaigaoka Hasama-cho, Yufu-shi, Oita-ken Japan; 2 Department of Medical Technology and Sciences, School of Health Sciences at Fukuoka, International University of Health and Welfare, Okawa, Japan; 3 Department of Biophysics, Faculty of Medicine, Oita University, Oita, Japan; 4 Department of Healthcare AI Data Science, Faculty of Medicine, Oita University, Oita, Japan

**Keywords:** Artificial intelligence, Chronic kidney disease, Machine learning, Predictive model, Renal replacement therapy

## Abstract

**Background:**

Predicting time to renal replacement therapy (RRT) is important in patients at high risk for end-stage kidney disease. We developed and validated machine learning models for predicting the time to RRT and compared its accuracy with conventional prediction methods that uses the rate of estimated glomerular filtration rate (eGFR) decline.

**Methods:**

Data of adult chronic kidney disease (CKD) patients who underwent hemodialysis at Oita University Hospital from April 2016 to March 2021 were extracted from electronic medical records (*N* = 135). A new machine learning predictor was compared with the established prediction method that uses the eGFR decline rate and the accuracy of the prediction models was determined using the coefficient of determination (R^2^). The data were preprocessed and split into training and validation datasets. We created multiple machine learning models using the training data and evaluated their accuracy using validation data. Furthermore, we predicted the time to RRT using a conventional prediction method that uses the eGFR decline rate for patients who had measured eGFR three or more times in two years and evaluated its accuracy.

**Results:**

The least absolute shrinkage and selection operator regression model exhibited moderate accuracy with an R^2^ of 0.60. By contrast, the conventional prediction method was found to be extremely low with an R^2^ of -17.1.

**Conclusions:**

The significance of this study is that it shows that machine learning can predict time to RRT moderately well with continuous values from data at a single time point. This approach outperforms the conventional prediction method that uses eGFR time series data and presents new avenues for CKD treatment.

**Supplementary Information:**

The online version contains supplementary material available at 10.1186/s12882-024-03527-9.

## Background

The number of dialysis patients is increasing globally and is expected to be 3.8 million people worldwide by 2021 [1]. Chronic kidney disease (CKD) is a concept proposed for the early detection of renal dysfunction, and it is estimated that 9.1% of the world’s population is affected by CKD [2]. The Kidney Disease Improving Global Outcomes (KDIGO) guideline provides a heat map of the risk of progression to end-stage kidney disease (ESKD), and the National Institute for Health and Care Excellence guideline recommends the kidney failure risk equation (KFRE) as a criterion for referral to a nephrologist [3–5]. However, CKD patients often delay referral to a nephrologist or discontinue seeing a nephrologist because of a lack of subjective symptoms and their reluctance to continue treatment.

In high-risk patients with ESKD, renal function often deteriorates progressively, making it crucial to predict the time to renal replacement therapy (RRT) to achieve a clearer and concrete description of the necessity of treatment. Conventionally, time series graphs of estimated glomerular filtration rate (eGFR) or reciprocal creatinine (Cr) are used to estimate the time to RRT based on the annual decline rate. However, these methods require time series data, which is difficult to generate for first-time patients. In addition, the method of reciprocal Cr has been reported to have low accuracy [6, 7].

Recently, there have been several reports on the application of artificial intelligence (AI) technology in medical care, covering a wide range of areas such as genomic medicine, image diagnosis, diagnostic and therapeutic support, and surgical support [8–12]. Machine learning is a technique for constructing a system to process tasks using big data. Supervised learning, which is a subcategory of machine learning, is divided into two types: classification, which predicts discrete values, and regression, which predicts continuous values. The classification accuracy in the field of image diagnosis is high, and the accuracy has already surpassed that of humans [13]. In risk assessment in the renal field, there were 39 reports on predicting the risk of developing acute kidney injury (AKI) as of March 2021, and Flechet et al. reported that the AKI predictor (available on the web) predicted AKI with a higher accuracy than that of physicians’ predictions [14, 15]. As of October 2021, there were 87 reports that predicted the risk of CKD patients developing ESKD within one to five years, with the area under the curve values ranging from 0.90 to 0.96, which indicates an accuracy comparable to that of the existing KFRE [16–20]. As an example of regression, Dai et al. developed a model to predict hospitalization costs for patients with CKD [21].

There have been several reports of classification models that can predict the risk of ESKD at a specific point in time; for example, “After 2 years, the probability of being ESKD is XX%.” However, no studies have reported a regression model that can predict the time to RRT with continuous values, such as “You will need RRT in XX days.” In this study, we use a regression model to predict the time to RRT with continuous values based on data obtained at a single time point. The proposed model can also be used for first time patients. Furthermore, we examine the accuracy of the conventional prediction method using eGFR time series data and compare it with that of the machine learning model.

## Methods

Adult patients (aged 20 years or older) with CKD who underwent hemodialysis at Oita University Hospital from April 2016 to March 2021 were selected retrospectively from electronic medical records. These patients were monitored at our hospital for at least three months until the induction of hemodialysis. Patients who had previously undergone RRT other than hemodialysis (peritoneal dialysis or renal transplantation) were excluded.

A total of 35 items were extracted from patient background and laboratory data up to the start of dialysis (Table 1). For laboratory data, we used CKD treatment items (anemia, CKD-MBD, blood glucose, and lipids) and indicators of nutritional status (albumin, total lymphocyte count (TLC), and cholinesterase (ChE)). These items have been used in previous studies on predictive models or noted in CKD guidelines as being associated with CKD progression [3, 17, 22–26]. For eGFR, we used the glomerular filtration rate estimation formula frequently used in Japan [27]:

*eGFR (ml/min/1.73 m* ^*2*^ *) = 194 × Cr* ^*− 1.094*^ *× age* ^*− 0.287*^ *(multiply by 0.739 for women).*

**Table 1:** Survey items (35 items)

Data types	Items
Patient background	age*, sex*, height*, weight*, CKD etiology*
Laboratory data	red blood cell (RBC)*, hemoglobin (Hb)*, hematocrit (Ht)*, mean corpuscular volume (MCV)*, mean corpuscular hemoglobin concentration (MCHC)*, total lymphocyte count (TLC), albumin(Alb)*, choline esterase (ChE), uric acid (UA), blood urea nitrogen (BUN)*, creatinine (Cr)*, estimated glomerular filtration rate (eGFR)*, sodium (Na)*, potassium (K)*, chlorine (Cl)*, calcium (Ca) *, phosphorus (P) *, glucose (Glu), hemoglobin A1c (HbA1c), triglyceride (TG), high-density lipoprotein cholesterol (HDL-C), low-density lipoprotein cholesterol (LDL-C), ferritin, iron (Fe), unsaturated iron binding capacity (UIBC), intact parathyroid hormone (i-PTH), hydrogen carbonate ion (HCO3-), urinary occult blood (UOb), urinary protein to creatinine ratio (UP/UCr)*, estimated urinary salt excretion (UNaCl)

*Final 20 items used for analysis

Data were processed and analyzed using Microsoft Excel 2021 and Python (version 3.8.16) from Google Colaboratory. The following packages were used in Python: Scikit-Learn (version 1.4.0) and Statsmodels (version 0.14.1). As an exploratory data analysis, the missing values and correlation coefficients were examined, and the data series with missing data were excluded from the analysis. Removal of missing data is necessary for the analysis, but it reduces the overall data volume and may introduce data bias. Categorical variables were converted to numeric values; CKD etiology was a 5-item categorical variable, including diabetic nephropathy (DN), with an additional data column created for each item and converted to 0 or 1 (one-hot encoding). This approach homogenizes the information and tends to increase the number of data items. The other items were continuous variables and were standardized.

Using Scikit-Learn’s GroupShuffleSplit function, the data were randomly split, with 75% for training and 25% for validation, such that data from the same case were not included in either group. Using the training data, supervised learning was performed with the number of days from the date of examination to the start of dialysis as the objective variable and the other items as explanatory variables. The learning algorithms used were linear regression, ridge regression, least absolute shrinkage and selection operator (LASSO) regression, elastic net, random forest, and gradient boosting decision tree (GBDT) based on the cheat sheet in Scikit-Learn [28]. Using Scikit-Learn’s GroupKfold function, the training data were divided randomly into four groups such that data from the same case were not included in the same group. Then, using Scikit-Learn’s GridSearchCV function the hyperparameters were adjusted by a grid search using cross-validation [29]. Hyperparameters control model performance and can be adjusted during model development to improve accuracy or address overfitting [30].

The coefficient of determination (R^2^) and mean absolute error (MAE) were used in previous reports to evaluate the accuracy of the regression model [16, 31, 32].

$$ {R}^{2}=1-\frac{\sum _{i=1}^{n}{\left({y}_{i}-{f}_{i}\right)}^{2}}{\sum _{j=1}^{n}{\left({y}_{j}-\stackrel{-}{y}\right)}^{2}},$$

$$ MAE=\frac{1}{n}{\sum }_{i=1}^{n}\left|{{y}_{i}-f}_{i}\right|,$$

where $$ {y}_{i}$$, $$ {f}_{i}$$, $$ \stackrel{-}{y}$$, and *n* represent the measured value, predicted value, average of measured values, and number of samples, respectively. R^2^ assumes a value of 1.0 or less, and the closer it is to 1.0, the higher is the accuracy. R^2^ does not mean square, as can be seen from the above definition, and it can be negative if the accuracy is extremely low [32]. The closer the MAE is to 0, the better is the model. In this study, R^2^ and MAE were calculated using the validation data to verify the accuracy of the model. In machine learning models, the smaller the difference in accuracy between the training data and validation data, the higher is the generalization performance. If the accuracy on the validation data is lower than that on the training data, the model is considered to be specialized for the training data and has low generalization performance; this condition is known as overfitting [29]. A learning curve shows the validation and training score of an estimator for varying numbers of training samples. It is a tool to determine the benefit derived from adding more training data and whether the estimator suffers more from underfitting or overfitting [33, 34]. In this study, we evaluated the generalization performance by comparing the R^2^ values on the training and validation data and by creating a learning curve.

We selected patients from the participants who were followed for more than two years and had a minimum of three eGFR measurements to examine the accuracy of the conventional prediction method using the eGFR decline rate. We calculated the eGFR decline rate at each time point using the SLOPE function (based on the least squares method) in Excel, referring to previous reports [35–37]. According to the guidelines of the Japanese Society for Dialysis Therapy, the number of days that the eGFR was estimated to be less than eight from each time point was used as the predicted value [38].

This study was approved by the Ethics Committee of Oita University, Faculty of Medicine (approval No. 2139: 2021). Additionally, because this is a retrospective study, the committee also approved the waiver of written informed consent and the adoption of the opt-out method. Information was disclosed on the website of the Department of Endocrinology, Metabolism, Rheumatology and Nephrology, Faculty of Medicine, Oita University.

## Results

**Fig. 1:**
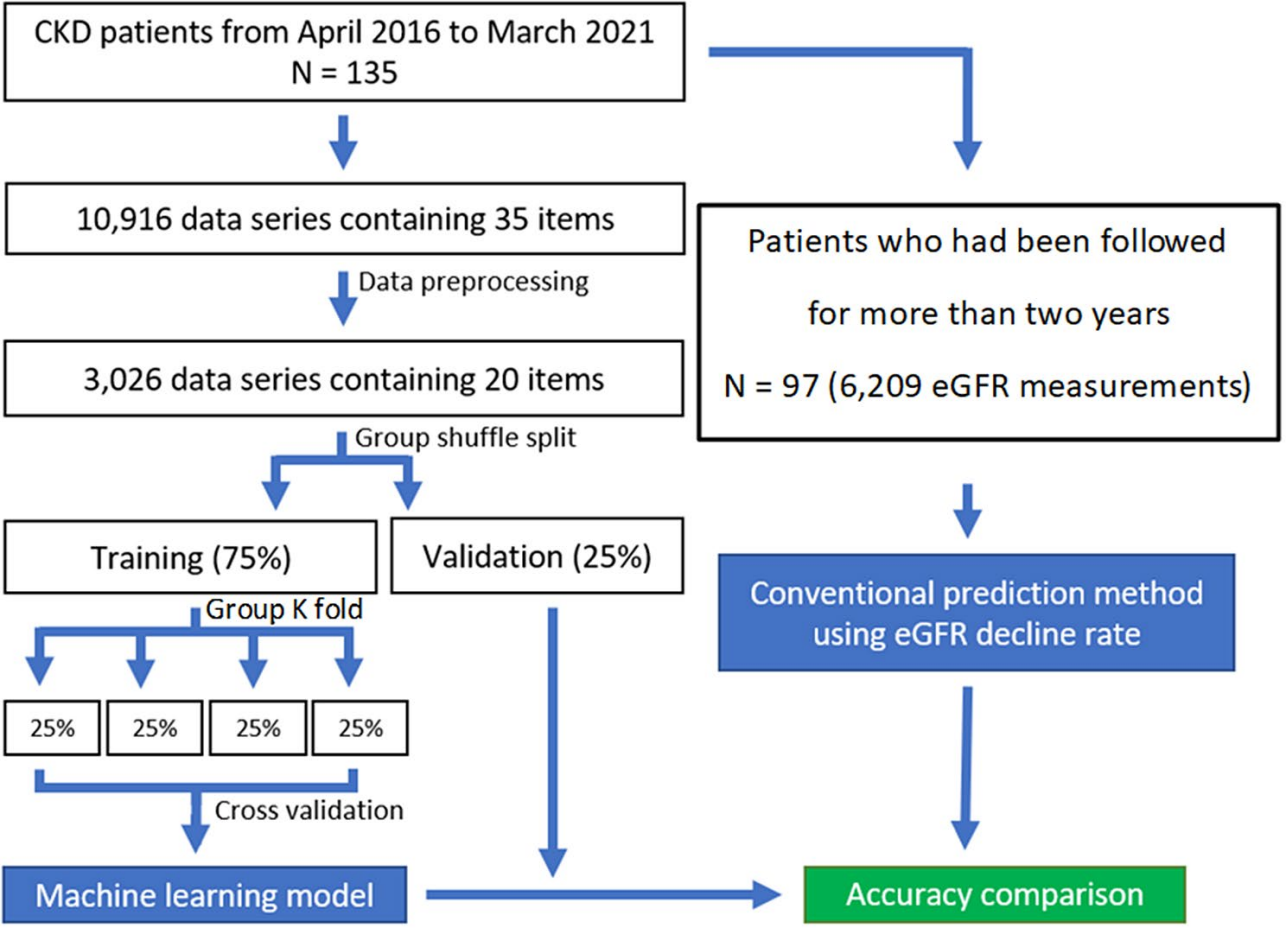
Flowchart from patient enrollment to model evaluation

Figure 1 shows the flowchart from patient enrollment to model evaluation. A total of 135 patients met the criteria. Hemodialysis cases were selected for this study, and patients who had previously started other renal replacement therapies were excluded: only one patient was on peritoneal dialysis and no patient underwent post renal transplant. The patient characteristics are listed in Table 2. The median age at the induction of dialysis was 71 years, median observation period was 496 days, most common CKD etiology was DN, and median laboratory findings at induction were Cr 7.5 mg/dL and eGFR 6.3 ml/min/1.73 m^2^. The details of the exploratory data analysis and machine learning are provided in the supplemental materials. A total of 10,916 data series containing 35 items were obtained from all patients. The number of missing data points for the survey items was examined, and items with numerous missing data points, such as bicarbonate ions and glucose, were difficult to use for the analysis (Supplementary Table S1). For the survey items, the Pearson’s correlation coefficient exhibited a correlation between renal function-related items and time to RRT, with a positive correlation for eGFR and a negative correlation for blood urea nitrogen (BUN) and Cr (Supplementary Figures S1–S2, Supplementary Table S2). Calcium, phosphorus, urinary protein/creatinine ratio, and albumin exhibited extremely weak correlations with time to RRT. These items have been used in prediction equations such as KFRE and were expected to be useful in this study. Among the items, very strong correlations were observed for red blood cells (RBCs), hemoglobin (Hb), and hematocrit (Ht), which are related to anemia, and when the variance inflation factor (VIF) was examined, multicollinearity was suspected for these items (Supplementary Tables S3–S4).

**Table 2:** Patient characteristics (*N* = 135)

Characteristics	n (%) or median (IQR)
Age (years)	71 (60–79)
Sex	
Male	87 (64%)
Female	48 (36%)
Observation period (days)	496 (160–1207)
CKD etiology	
Diabetic nephropathy (DN)	52 (38%)
Nephrosclerosis (NS)	42 (31%)
Chronic glomerulonephritis (CGN)	17 (12%)
Polycystic kidney disease (PKD)	5 (3%)
Other	19 (16%)
Comorbidities	
Diabetes mellitus	60 (44%)
Hypertension	130 (96%)
Dyslipidemia	97 (72%)
Hyperuricemia	70 (52%)
Heart failure with reduced ejection fraction	9 (7%)
Ischemic heart disease	12 (9%)
Cerebrovascular disease	16 (12%)
Peripheral vascular disease	5 (4%)
Medication at initial visit	
Renin-angiotensin system inhibitors	95 (70%)
Mineralocorticoid-receptor antagonists	9 (7%)
Sodium-glucose cotransporter 2 inhibitors	4 (3%)
Glucagon-like peptide-1 receptor agonist	8 (6%)
Statin	81 (22%)
Uric acid-lowering drug	69 (51%)
AST-120	30 (22%)
Erythropoiesis stimulating agent	18 (13%)
Sodium bicarbonate	7 (5%)
Cr at RRT initiation (mg/dL)	7.3 (6.0–8.7)
eGFR at RRT initiation (ml/min/1.73 m^2^)	6.2 (4.7–7.6)

Categorical variables are presented as *n* (%) and continuous variables as median (IQR).

N: total number of people, n: number of items, IQR: interquartile range

**Fig. 2:**
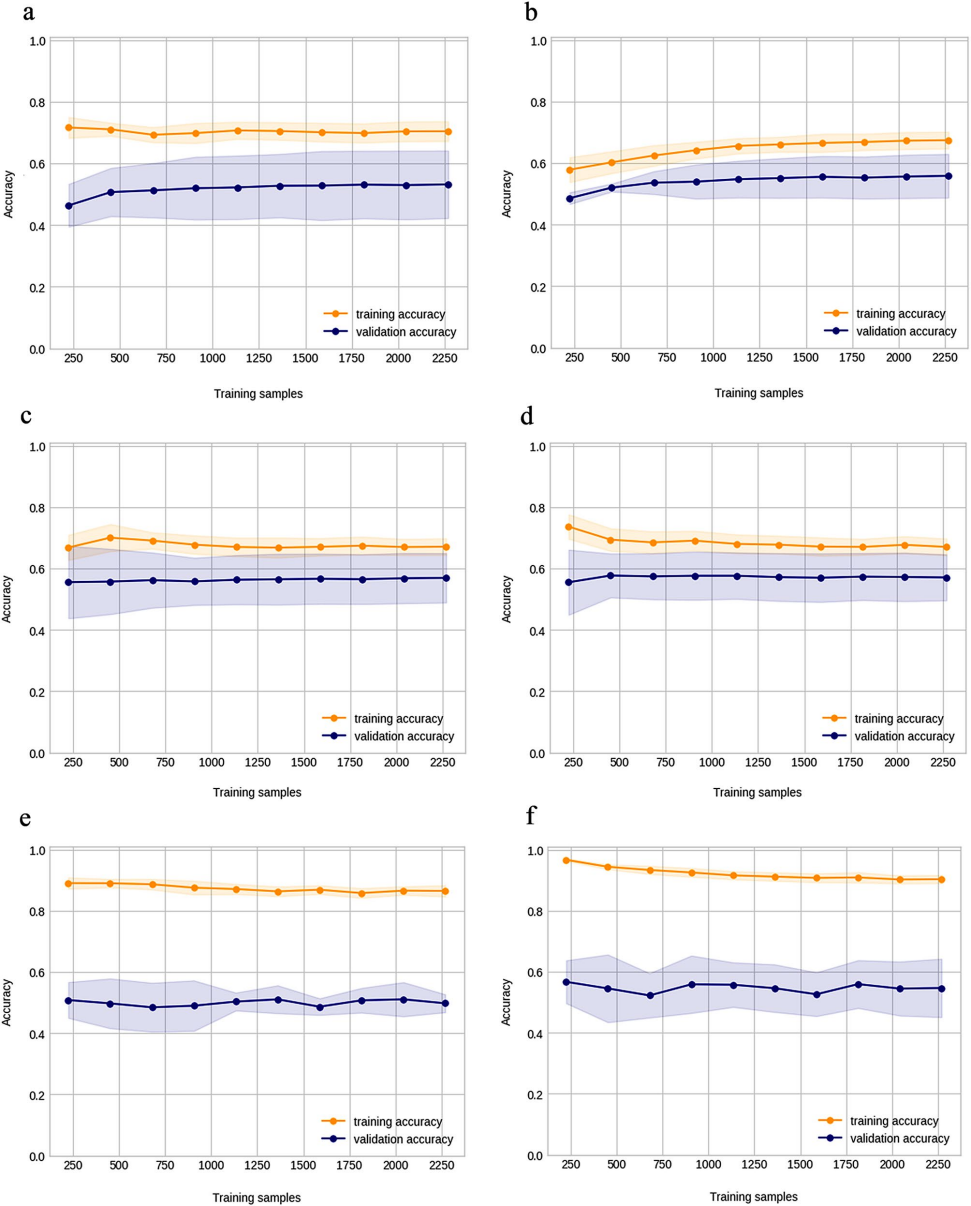
Learning curves for each algorithm.(**a**) Linear regression, (**b**) Ridge regression, (**c**) LASSO regression, (**d**) Elastic net, (**e**) Random forest, and (**f**) GBDT. LASSO regression shows that the accuracy of the training data and the validation data converge to a close value as the number of samples increases. On the other hand, in the GBDT, the accuracy of the training data and the validation data remain divergent even as the number of samples increases

**Fig. 3:**
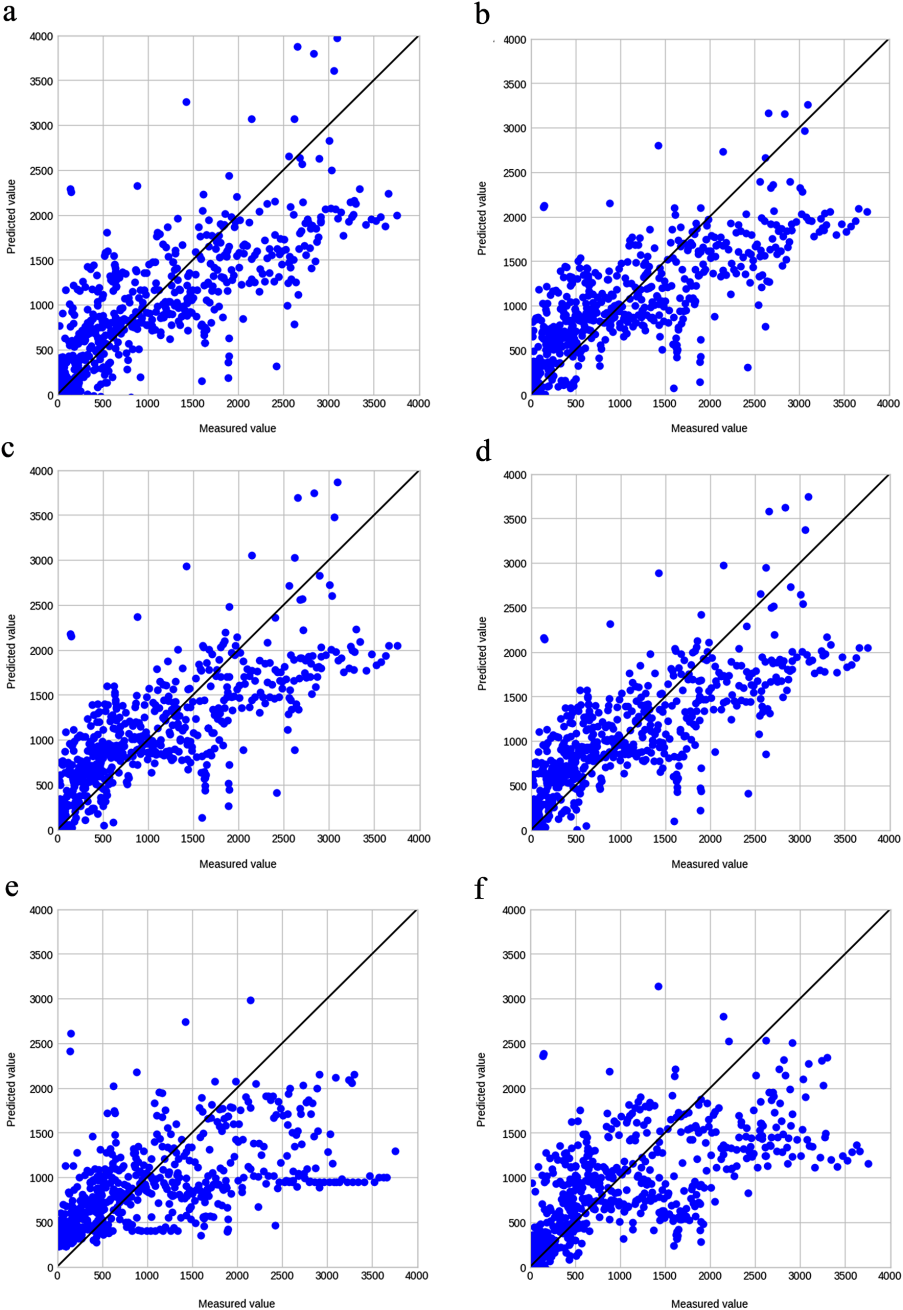
Relationship between predicted and measured values for each algorithm. (**a**) Linear regression, (**b**) Ridge regression, (**c**) LASSO regression, (**d**) Elastic net, (**e**) Random forest, and (**f**) GBDT. For all algorithms, the error in the predictions tended to increase as the measured values increased

Data series with missing values were excluded in preprocessing. The items were reduced stepwise in the preliminary study because this method reduces the overall number of data series when more items are used. Finally, the highest accuracy was achieved using 3,026 data series containing 20 items (shown in Table 1). The training algorithm and cross-validation results for this dataset are summarized in Table 3 and the hyperparameters in Supplementary Table S5. In LASSO regression, the R^2^ for cross-validation was 0.59 with moderate accuracy, and the R^2^ difference between cross-validation and training was small compared to those of other algorithms. The MAE was 488, implying that the model had a mean error of 488 days. Conversely, ensemble models such as GBDT appeared to be overfitting, with large R^2^ differences between cross-validation and training. The learning curves visually confirmed the overfitting, showing that LASSO converged to the same value for the training and validation data (Fig. 2-c), whereas GBDT converged with a divergence between the two (Fig. 2-f). Table 4 summarizes the results of validating the accuracy of the created models on the validation data. LASSO regression exhibited an R^2^ of 0.60, which is as stable as that of the cross-validation results, while GBDT exhibited an R^2^ of 0.51, which is lower than that of the cross-validation results. GBDT was overfitting, which may have reduced its accuracy on unknown data. Figure 3 shows a scatter plot of the relationship between the predictions and the measured values for the validation data. The larger the measured values, the larger was the deviation from the predictions.

**Table 3:** Cross-validation results

Algorithm	R^2^ (training fold)	MAE (training fold)	R^2^ (cross-validation fold)	MAE (cross-validation fold)
Linear regression	0.72	418	0.50	531
Ridge regression	0.68	443	0.56	509
LASSO regression	0.69	427	0.59	488
Elastic net	0.68	439	0.58	500
Random forest	0.86	294	0.58	484
GBDT	0.93	198	0.62	459

**Table 4:** Validation data results

Algorithm	R^2^ (validation data)	MAE (validation data)
Linear regression	0.55	471
Ridge regression	0.56	471
LASSO regression	0.60	450
Elastic net	0.58	463
Random forest	0.48	485
GBDT	0.51	440

LASSO is a linear regression model that uses L1 regularization. A linear regression model is represented by the prediction equation *y = a*_*1*_*  x  *_*1*_ *+ a*_*2*_*  x  *_*2*_*+… + a*_*n*_*x*_*n*_*+ b*, where *y*, *x*, and *a* represent the objective variable (predicted value), explanatory variable (data items), and regression coefficient, respectively. LASSO can automatically select explanatory variables by adjusting the regression coefficients with L1 regularization. Regression coefficients for the LASSO regression are listed in Table 5, with eGFR having the largest value and highest contribution to the prediction, and the coefficients for several items, such as RBC being 0, implying no contribution to the prediction.

**Fig. 4:**
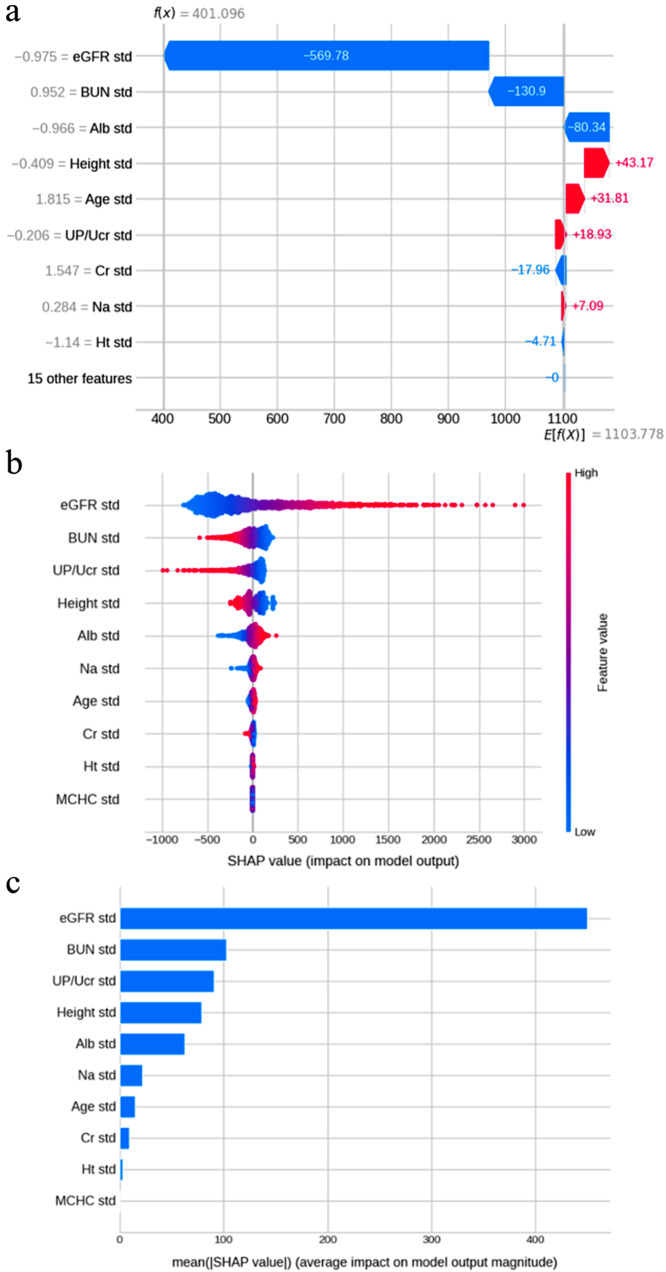
SHAP value(**a**) Waterfall plot of a randomly selected case. The bottom of the waterfall plot begins with the expected value of the model output, each row shows the positive (red) or negative (blue) contribution of each item, and the top shows the final output. This patient already had severe renal dysfunction, and the expected value was 1,234.956; however, the final output was 401.096 because there were changes such as a standardized eGFR of − 695.53 and a standardized BUN of − 139.3. (**b**) Summary plot (scatter plot) of all cases analyzed in this study. As shown at the top of the plot, the larger and redder the eGFR feature, the larger is the positive contribution (SHAP value) to prediction, which indicates a positive correlation. (**c**) Summary plot (bar chart) of the average absolute SHAP values for all cases, which shows that eGFR had the greatest influence

After running the prediction, SHAplay Additive exPlanations (SHAP) can be used to visualize the impact of each item on the prediction [39]. The waterfall plot provides an explanation of the predicted results for each individual case. Figure 4-a is a waterfall plot of a randomly selected case that specifically shows the output of predicted values by eGFR, BUN, and other inputs. Figure 4-b is a summary plot (scatter plot) for all cases analyzed in this study, and Fig. 4-c is a summary plot (bar chart) of the average SHAP absolute value for all cases. The summary plot also shows a high contribution of eGFR. In this way, SHAP is useful for interpreting predictions not only for individual cases but also for entire cases.

**Table 5:** Regression coefficients for LASSO regression

Explanatory variable	Regression coefficients
Age	17.6
Sex	0
Height	-95.0
Weight	0
CKD etiology	0
RBC	0
Hb	0
Ht	3.7
MCV	0
MCHC	0
Alb	80.8
BUN	-126.5
Cr	-11.5
Na	31.8
K	0
Cl	0
Ca	0
P	0
eGFR	551.0
UP/UCr	-130.0

The small number of cases in this study did not allow for sufficient subgroup analysis by CKD etiology or stage. However, when reanalyzing only the data series with DN as the CKD etiology in the LASSO regression, the R^2^ was 0.60 and MAE was 302 in 1096 data series, indicating a decrease in MAE. Elsewhere, when reanalyzed only in the KDIGO CKD heatmap high-risk data series with LASSO regression, the R^2^ was 0.62 and MAE was 396 in the 3025 data series, indicating an increase in R^2^. In addition, we reduced the number of items with suspected multicollinearity and items with small contributions to prediction by referring to the correlation matrix, VIF, regression coefficient, and SHAP values described, and we reanalyzed them with 12 items (age, sex, height, weight, CKD etiology, Hb, albumin, sodium, potassium, chlorine, eGFR, and urinary protein to creatinine ratio.) LASSO regression had an R^2^ of 0.59 and MAE of 407, indicating a slight decrease in accuracy.

**Fig. 5:**
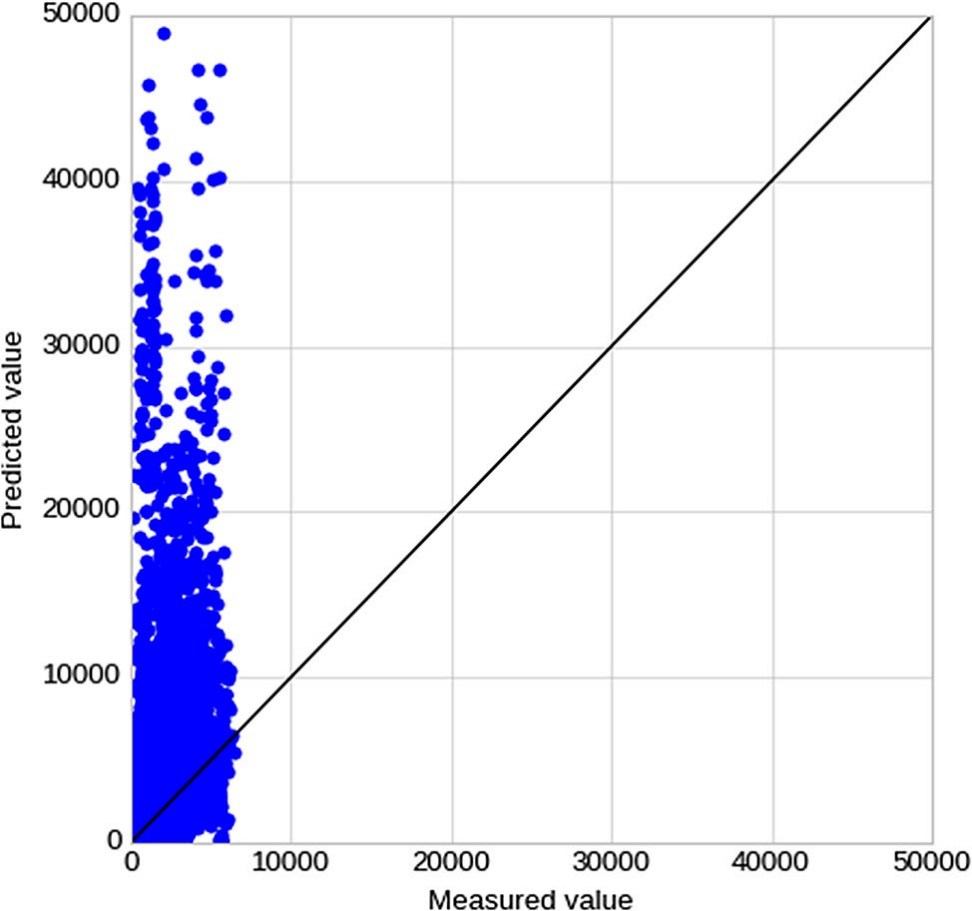
Relationship between predicted and measured values for the conventional eGFR time series method.Predictions tended to be very large, with a maximum predicted value of 73,549, which is considered an outlier, and for readability, the Y-axis is shown with a range up to 50,000

For the conventional prediction method that uses the rate of eGFR decline, 97 patients met the criteria, with a total of 6,209 eGFR measurements. A scatter plot of the relationship between the predicted and measured values is shown in Fig. 5. The predicted values tended to be larger than the measured values. The accuracy was R^2^ = − 17.1 and MAE = 2466, indicating an extremely low prediction accuracy.

## Discussion

A notable feature of this study is that, unlike existing ESKD risk, we focused on predicting time to RRT using continuous values and created a moderately accurate prediction model. This model can predict the time to RRT (RRT start date) based on data obtained at a single time point, and therefore it can provide concrete information even for first-time patients and clearly indicate the need to start treatment. As conditions change over time after intervention, the repeated prediction model can be used to predict the RRT start date each time. If the predicted RRT start date is extended, the patient will realize the benefits of treatment and will be more motivated to continue treatment. Even in the unfortunate case that the predicted RRT start date is moved up, it may be helpful to identify the reason for this change. Planned dialysis induction has a better prognosis [40]; if machine learning can predict the RRT start date, it will enable planned therapy selection and access construction. In addition, because the start of dialysis has a significant impact on a patient’s life, predicting the RRT start date is useful for the patient’s own life planning. The prediction of time to RRT based on regression may be a more patient-oriented outcome than the prediction of the risk of ESKD based on classification. For other progressive diseases, e.g., in the case of malignant tumors, prognosis and treatment efficacy are discussed in terms of a five-year survival rate (risk) in the early stages; however, the life expectancy (time) is often considered in advanced stages or when treatment is difficult. Numerous methods have been reported to predict life expectancy in days rather than risk [41, 42]. In the case of CKD, life can be maintained with RRT; however, the lifestyle needs to be changed drastically. Therefore, the argument of predicting time to renal death in CKD may be useful, at least in the cases of high risk for ESKD. The number of elderly CKD patients with complications has increased in recent years, causing the concept of conservative kidney management to emerge [43]. The indication for renal biopsy, immunosuppressive therapy, and RRT should be determined based on the prognosis for time to renal death and complications, and in this regard, prediction of time to RRT is important. Some people become depressed when they are told how long it will take to reach RRT, and therefore care must be taken in actual use. However, it is expected to be a useful tool to realize better treatments of CKD when used effectively.

On the other hand, the accuracy of the conventional prediction method using the eGFR decline rate was extremely low. As illustrated in Fig. 3-b, the predicted values tended to be larger than the measured values. The difference between the measured and predicted values would be small if the eGFR decline rate is constant during the observation period; however, the predicted values would be larger than the measured values if the eGFR decline rate increased with time. For instance, in diabetic nephropathy, which was the most common etiology in this study, the eGFR decline rate increased after the appearance of a urinary protein, as shown in Supplementary Figure S3 [44, 45]. In this case, the eGFR decline rate was small in the initial stage, and the time to RRT was predicted to be long; however, if the eGFR decline rate increased during the course, the time to RRT became shorter than that during the initial prediction. Considering a nonlinear approximate curve instead of a linear regression may be necessary; however, it is difficult to apply a constant rule because the rate of decline varies for each case. The method using the decline rate of the reciprocal Cr cannot be used unless it is limited to patients with advanced CKD whose renal function worsens in a linear manner. Even in that case, this method suffers from an error of approximately one year [6]. Although time series information on renal function is important, it seems difficult to predict time to RRT based on the eGFR decline rate alone.

In this study, 10,916 data points were extracted from 135 cases and analyzed, assuming that the data were independent; however, data from the same cases are not completely independent and data bias is likely to occur. Data used in machine learning models should be independent and identically distributed for training and validation [46]. When using multiple time series data from the same case, as in this study, it is necessary to ensure that data from the same case do not leak into both the training and validation groups. In this study, data from the same cases were grouped together to address leakage, but other methods may also be useful, like dividing data by the time axis [47, 48]. The learning models used in this study were linear models: linear regression, ridge regression, LASSO regression, and elastic net, and nonlinear models: random forest and GBDT. In general, the use of nonlinear models is expected to increase accuracy when the linear regression model does not adequately represent the characteristics of the data. With reference to the cheat sheet in Scikit-Learn, the aforementioned models were used in this study. However, in this case, the nonlinear model tended to overfit even after hyperparameter adjustment and cross-validation, and its accuracy on validation data was low. By contrast, the LASSO model, a linear model, exhibited low overfitting tendency and stable accuracy on validation data. Overfitting countermeasures include increasing the training data, simplifying the model by adjusting hyperparameters, cross-validation, regularization, and bootstrapping [49–51]. LASSO is an algorithm that can reduce the number of variables through regularization, which may have led to stable results. The small number of cases and the relatively large number of feature variables may have caused the nonlinear model to overfit [34]. However, in this study, the LASSO model has a limitation in that regularization prevents the incorporation of useful information such as the CKD etiology. In fact, subgroup analysis in DN has improved accuracy, and if the number of cases is increased and analyzed by etiology in future, the overall accuracy of the model may be improved. Other general machine learning issues include multicollinearity, which is often a problem when using multiple regression models in statistics [52, 53]. Multicollinearity is a problem in which the predictors are correlated, and creating a model with multicollinear items makes it impossible to estimate the contribution by the regression coefficient. Multicollinearity is estimated using correlation matrices and VIF, and it is common to remove multicollinear items from the model. However, in machine learning, methods such as regularization and principal component analysis have been used to reduce the effect of multicollinearity, and good results have been obtained [54]. In this study, multicollinearity was suspected in anemia-related items, and variables were selected by regularization using LASSO regression. The accuracy decreased slightly when we used the conventional method of removing items with multicollinearity. In addition, there is concern that machine learning creates complex predictive models that cannot be interpreted easily by humans, making it a black box. The explainability of predictive models and the interpretability of predictions are especially important when used in medical applications where decisions can be life-threatening [55]. LASSO regression uses the regression coefficients to explain the contribution of the explanatory variables in the model. In the present model, eGFR contributes the most, indicating that the model reflects the results of eGFR to a large extent, which is a reasonable result. In addition, after running the predictions, SHAP can be used to visualize the actual contribution of the explanatory variables to the predictions [39, 55, 56]. In the case shown in Fig. 4-a, it is understandable that inputs such as eGFR and BUN had a significant impact on the number of days predicted. When used in actual practice, the machine learning model can be applied to display the predicted number of days to RRT and the contribution of these items. Although the model is not designed for causal inference in this case, it could be clinically useful to examine the items that the AI focused on to make its predictions.

Limitations of this study include the fact that it was a single-center study, the regional nature of the cases, the small number of cases compared to previous studies, and the concern about data bias due to this. In addition, although only hemodialysis cases were included in this study, if peritoneal dialysis and renal transplantation cases can be added to the analysis in the future, it may be possible to apply this study to more general CKD cases. Owing to the small sample size, it was difficult to adequately analyze subgroups by etiology, stage, blood pressure, and glucose control status of CKD. The etiology and high-risk cases of CKD may have different modes of renal function deterioration, and in this study, increased accuracy was obtained in the analysis of DN and high-risk cases. Reanalysis with more cases is desirable in future. In addition, it was difficult to extract drug data at each time point. Validation on an external cohort is also an issue for the future. Another limitation of the machine learning model in this study is that, unlike the eGFR time series method, it does not incorporate time series information. The model calculates the same predicted value if the clinical data at the time of prediction are similar, regardless of whether renal function has been stable for several years or has deteriorated rapidly. Presently, it is safe to use this model in combination with the eGFR time series method, and the incorporation of time series information is an issue for the future. However, there have been no similar reports to date. We believe that this study is useful because it represents a potential new patient-based outcome.

## Conclusions

A moderately accurate prediction model was developed by using a machine learning regression model to predict time to RRT with continuous values using data obtained at a single time point. This approach yielded better results than the conventional prediction method that uses eGFR time series data. The ability to specify the time to RRT is useful not only for medical practitioners to make treatment decisions but also for patients to motivate themselves to undergo treatment and for life planning.

### Electronic supplementary material

Below is the link to the electronic supplementary material.

Supplementary Material 1

## Data Availability

The data underlying this article can be shared upon reasonable request to the corresponding author.

## Abbreviations

CKD: Chronic kidney disease, KDIGO:Kidney Disease Improving Global Outcomes
ESKD: End-stage kidney disease
KFRE: Kidney failure risk equation
RRT: Renal replacement therapy
eGFR: Estimated glomerular filtration rate
Cr: Creatinine
AI: Artificial intelligence
AKI: Acute kidney injury
TLC: Total lymphocyte count
ChE: Choline esterase
LASSO: Least absolute shrinkage and selection operator
GBDT: Gradient boosting decision tree
R^2^: Coefficient of determination
MAE: Mean absolute error
BUN: Blood urea nitrogen
VIF: Variance inflation factor
RBC: Red blood cell
SHAP: SHAplay Additive exPlanations
Hb: hemoglobin
Ht: hematocrit
MCV: Mean corpuscular volume
MCHC: Mean corpuscular hemoglobin concentration
Alb: Albumin
UA: Uric acid
Glu: Glucose
HbA1c: Hemoglobin A1c
TG: Triglyceride
HDL-C: High-density lipoprotein cholesterol
LDL-C: Low-density lipoprotein cholesterol
UIBC: Unsaturated iron binding capacity
i-PTH: Intact parathyroid hormone
HCO3-: Hydrogen carbonate ion
UOb: Urinary occult blood
UP/UCr: Urinary protein to creatinine ratio
UNaCl: Estimated urinary salt excretion
DN: Diabetic nephropathy
NS: Nephrosclerosis
CGN: Chronic glomerulonephritis
PKD: Polycystic kidney disease
IQR: Interquartile range
