# Supplementary material for: Development and validation of a machine learning model to predict time to renal replacement therapy in patients with chronic kidney disease
Source: BMC Nephrol. 2024 Mar 16;25:101. doi: 10.1186/s12882-024-03527-9 (PMC10943785; doi:10.1186/s12882-024-03527-9)
Supplement: Supplementary file 1 — Supplementary Material 1 [file 12882_2024_3527_MOESM1_ESM.docx]

**Supplementary material**

Supplementary Table S1. Number of data points and missing data points for each survey item

| Items | Number of data points  (Number of missing data points) | Items | Number of data points  (Number of missing data points) |
| --- | --- | --- | --- |
| Age | 10916 (0) | K | 9505 (1411) |
| Sex | 10916 (0) | Cl | 9338 (1578) |
| Height | 10916 (0) | Ca | 6810 (4106) |
| Weight | 10916 (0) | P | 6310 (4606) |
| CKD etiology | 10916 (0) | Glu | 57 (10859) |
| RBC | 9233 (1683) | HbA1c | 4785 (6131) |
| Hb | 9233 (1683) | TG | 6680 (4236) |
| Ht | 9233 (1683) | HDL-C | 5485 (5485) |
| MCV | 9233 (1683) | LDL-C | 4095 (6821) |
| MCHC | 9233 (1683) | ferritin | 1414 (9502) |
| TLC | 6551 (4265) | Fe | 2155 (8761) |
| Alb | 8463 (2453) | UIBC | 1946 (8970) |
| ChE | 3628 (7288) | i-PTH | 514 (10402) |
| UA | 8291 (2625) | HCO3- | 122 (10794) |
| BUN | 9605 (1311) | UOb | 4584 (6332) |
| Cr | 9651 (1265) | UP/UCr | 5185 (5731) |
| eGFR | 9651 (1265) | UNaCl | 2498 (8418) |
| Na | 9505 (1411) |  |  |

Supplementary Table S2. Pearson's correlation coefficients between survey items and time to RRT with absolute values greater than 0.3 (items are standardized)

| Items | Correlation coefficients |
| --- | --- |
| eGFR | 0.73 |
| Cr | −0.66 |
| BUN | −0.58 |
| Ca | 0.46 |
| P | 0.40 |
| UP/UCr | −0.34 |
| Alb | 0.30 |

Supplementary Table S3. Pearson's correlation coefficients between survey items with absolute values greater than 0.6 (items are standardized)

| Items |  | Correlation coefficients |
| --- | --- | --- |
| Hb | Ht | 0.96 |
| RBC | Ht | 0.92 |
| RBC | Hb | 0.90 |
| BUN | Cr | 0.76 |
| Cr | eGFR | −0.76 |
| HEIGHT | WEIGHT | 0.69 |
| SEX | HEIGHT | −0.69 |
| BUN | eGFR | −0.68 |
| Cr | P | 0.61 |

Supplementary Table S4. VIF of survey items (5 or more)

| Items | VIF |
| --- | --- |
| RBC | 141 |
| Hb | 788 |
| Ht | 864 |
| MCHC | 62 |
| MCV | 22 |
| Cr | 5.4 |

Supplementary Table S5. Hyperparameters of each algorithm's prediction model

| Algorithm | Hyperparameters |
| --- | --- |
| Linear regression | None |
| Ridge regression | Alpha 420 |
| LASSO regression | Alpha 36 |
| Elastic net | Alpha 15，l1_ratio 0.99 |
| Random forest | max_depth 5, max_features sqrt, max_leaf_nodes 20, min_samples_leaf 5, min_samples_split 10, n_estimators 100 |
| GBDT | 'learning_rate 0.1, max_depth 3, max_features log2, min_samples_leaf 1, n_estimators 80 |

**
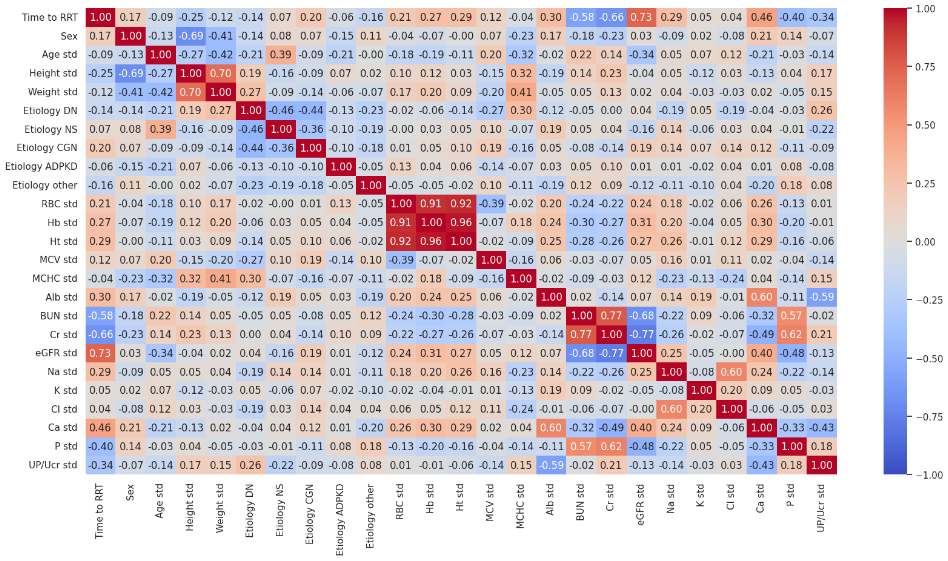
**

Supplementary Figure S1. Pearson's correlation coefficient heat map of survey items.


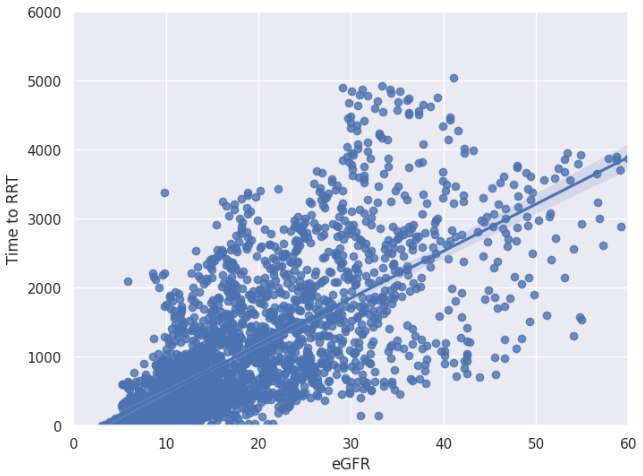


Supplementary Figure S2. Relationship between eGFR and time to RRT. (We used eGFR before standardization.)


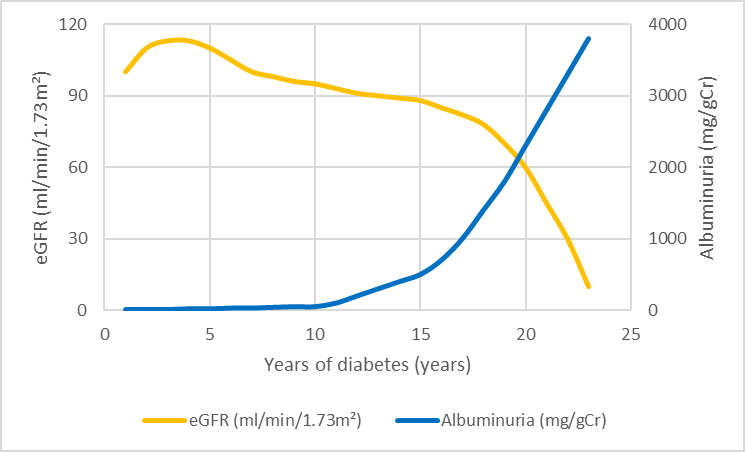


Supplementary Figure S3. Natural history of diabetic nephropathy in type 1 DM, as described by Mogensen et al [45].
